# Differential Expression of *BARD1* Isoforms in Melanoma

**DOI:** 10.3390/genes12020320

**Published:** 2021-02-23

**Authors:** Lorissa I. McDougall, Ryan M. Powell, Magdalena Ratajska, Chi F. Lynch-Sutherland, Sultana Mehbuba Hossain, George A. R. Wiggins, Agnieszka Harazin-Lechowska, Bożena Cybulska-Stopa, Jyoti Motwani, Erin C. Macaulay, Glen Reid, Logan C. Walker, Janusz Ryś, Michael R. Eccles

**Affiliations:** 1 Department of Pathology, Otago Medical School, Dunedin Campus, University of Otago, Dunedin 9010, New Zealand; lorissa.mcdougall@otago.ac.nz (L.I.M.); ryan.powell@postgrad.otago.ac.nz (R.M.P.); magda.ratajska@otago.ac.nz (M.R.); lynch781@student.otago.ac.nz (C.F.L.-S.); mehbuba.hossain@postgrad.otago.ac.nz (S.M.H.); mail.jyotim@gmail.com (J.M.); erin.macaulay@otago.ac.nz (E.C.M.); glen.reid@otago.ac.nz (G.R.); 2 Department of Biology and Medical Genetics, Medical University of Gdansk, 80-211 Gdansk, Poland; 3 Department of Pathology and Biomedical Science, University of Otago, Christchurch 8011, New Zealand; george.wiggins@otago.ac.nz (G.A.R.W.); logan.walker@otago.ac.nz (L.C.W.); 4 Department of Tumour Pathology, Maria Sklodowska-Curie National Research Institute of Oncology, Cracow Branch, 8011 Cracow, Poland; harazin@wp.pl (A.H.-L.); janusz.rys@onkologia.krakow.pl (J.R.); 5 Department of Clinical Oncology, Maria Sklodowska-Curie National Research Institute of Oncology, Cracow Branch, 8011 Cracow, Poland; bcybulskastopa@vp.pl; 6 Maurice Wilkins Centre for Molecular Biodiscovery, Auckland 1010, New Zealand

**Keywords:** melanoma, *BARD1*, nanopore sequencing, RNA-Seq

## Abstract

Melanoma comprises <5% of cutaneous malignancies, yet it causes a significant proportion of skin cancer-related deaths worldwide. While new therapies for melanoma have been developed, not all patients respond well. Thus, further research is required to better predict patient outcomes. Using long-range nanopore sequencing, RT-qPCR, and RNA sequencing analyses, we examined the transcription of *BARD1* splice isoforms in melanoma cell lines and patient tissue samples. Seventy-six *BARD1* mRNA variants were identified in total, with several previously characterised isoforms (γ, φ, δ, ε, and η) contributing to a large proportion of the expressed transcripts. In addition, we identified four novel splice events, namely, Δ(E3_E9), ▼(i8), IVS10+131▼46, and IVS10▼176, occurring in various combinations in multiple transcripts. We found that short-read RNA-Seq analyses were limited in their ability to predict isoforms containing multiple non-contiguous splicing events, as compared to long-range nanopore sequencing. These studies suggest that further investigations into the functional significance of the identified *BARD1* splice variants in melanoma are warranted.

## 1. Introduction

Melanoma develops following the malignant transformation of pigment-producing melanocytes and is the fifth most common form of cancer in adults [1]. Importantly, while melanoma is responsible for the majority of skin cancer-related deaths, it accounts for <5% of cutaneous malignancies [2]. Over the past decade, great progress has been made in treating unresectable and metastatic melanomas. Indeed, five-year survival rates have increased from ~5% a decade ago to ~50% for those treated with combination immunotherapies, and ~33% for combination BRAF/MAPK kinase inhibitors [1]. Despite these tremendous advances, not all patients respond well to approved treatments. Moreover, the incidence of cutaneous melanoma is increasing at a startling 4–6% per annum [3]. Thus, there is a great need to further investigate melanoma cell biology to increase our understanding of long-term response and treatment outcomes. 

*BARD1* (BRCA1-Associated RING Domain 1; 2q34-q35) was initially discovered as a BRCA1-binding partner [4]. The interaction between RING domains within *BARD1* and BRCA1 is critical for the tumour suppressive effects of the protein heterodimer, stimulating ubiquitin ligase activity [5]. The identified substrates for ubiquitination by the *BARD1*–BRCA1 heterodimer include the histone proteins, H2A, and the H2AX variant, γ tubulin, and subunits of the RNA polymerase 2 holoenzyme [6,7,8]. As such, the heterodimer is implicated in the regulation of multiple cellular processes, including nucleosome remodelling, transcription, centrosome formation, and cell cycle progression. Moreover, the heterodimer’s function is crucial for cell survival and regulating DNA damage responses [9]. Under prolonged stress, *BARD1* is capable of inducing apoptosis via interactions with TP53 [10]. 

The role of *BARD1* in limiting tumorigenesis has been highlighted in studies of neuroblastoma, where single nucleotide polymorphisms (SNPs), which increase full-length *BARD1* mRNA (*BARD1*-FL) expression, hinder tumour progression [11]. Conversely, SNPs associated with decreased *BARD1*-FL expression were associated with higher-risk neuroblastoma [12]. Despite the recognition of *BARD1*’s role in limiting tumorigenesis, oncogenic mutations within *BARD1* are infrequent, especially when compared to that of *BRCA1* [13]. This observation has spurred much interest into investigating novel splice variants of *BARD1*. Traditionally, splice variants refer to exon-modifying events at the level of the RNA, while isoforms define the subsequent changes that may be carried through to the final protein structure [14]. In addition to the 11-exon containing *BARD1*-FL, numerous splice isoforms have been identified thus far, including *BARD1*-α, -β, -γ, -δ, -ε, -η, and -ω [15,16]. Interestingly, *BARD1* splice isoforms, which lack the RING domain, such as *BARD1*-β and -δ, show effects that are antagonistic to that of *BARD1*-FL, and have been associated with progression and poor prognosis in multiple cancer types [17,18,19]. Moreover, it has been suggested that increased prevalence of *BARD1* isoforms over *BARD1*-FL promotes tumorigenesis [20]. 

Currently, investigations into *BARD1* splice isoforms in melanoma are not present in the literature. However, due to the recognition of *BARD1*’s pleiotropic roles in DNA repair processes, in addition to the high mutation burden commonly observed in melanoma, we hypothesised a pro-tumorigenic role for alternate *BARD1* isoforms in melanoma [21,22,23,24,25,26].

Herein, we present the first study assessing the expression of *BARD1* mRNA splice isoforms in melanoma cell lines and archival formalin-fixed paraffin-embedded (FFPE) tissue samples. The results show that previously identified *BARD1* mRNAs encoding isoforms, as well as novel splice isoforms, can be detected using both long-range nanopore sequencing, in addition to short-read and targeted approaches, including RNA-Seq and RT-qPCR in both melanoma cell lines and tissue samples. Due to the nature of the read lengths provided by nanopore sequencing, the technique provides the most comprehensive analyses of splice isoforms. Comparison of the aforementioned RNA sequencing technologies are reviewed elsewhere [27,28,29].

## 2. Materials and Methods 

### 2.1. NZM and Melanocyte Cell Lines 

The 12 New Zealand melanoma (NZM) cell lines used for this study were generated from metastatic melanoma, as described previously [30]. Written informed consent was obtained from all patients under the Auckland Area Health Board Ethics Committee guidelines. The NZM cell lines were maintained in α-MEM media (Gibco, ThermoFisher Scientific, Waltham, MA, USA) supplemented with 10% FCS (Moregate, Bulimba, QLD, Australia) and 1% penicillin–streptomycin (Gibco, ThermoFisher Scientific, Waltham, MA, USA). Cell cultures were passaged twice at 80% confluency prior to harvesting for RNA extraction using the appropriate kit (QIAGEN, Germantown, MD, USA).

The melanocyte cell lines used as a non-malignant control were commercial cell lines isolated from human epidermal melanocytes of neonates (HEMnLP) or adult (HEMaLP) tissue. The melanocyte cell line Mel-ST (immortalised using SV40ER and hTERT) was used for nanopore sequencing.

The RNA was extracted using an RNeasy Mini Kit (QIAGEN, Germantown, MD, USA). The quantity and quality of isolated RNA was determined using a NanoPhotometer (Implen, Munich, Germany) and a Qubit Fluorometer (ThermoFisher Scientific, Waltham, MA, USA). For reverse transcription, 20 ng/µL (totalling 200 ng per reaction) of total RNA was used, according to the manufacturer’s instructions (High-Capacity cDNA RT kit, ThermoFisher Scientific). The NZM cell lines had been authenticated by the Auckland Cancer Society Research Centre.

### 2.2. Archival Formalin-Fixed Paraffin-Embedded (FFPE) Tissue Blocks 

From the biobank at the Department of Tumour Pathology, Maria Sklodowska-Curie National Research Institute of Oncology, Krakow, Poland, 18 metastatic melanoma tissue sample blocks were selected. In order to minimise sample heterogeneity, tissue macro-dissection was performed. RNA was extracted using the FormaPure XL Total Kit (Beckman Coulter, Brea, CA, USA). The quantity and quality of isolated RNA was determined using a NanoPhotometer (Implen, Munich, Germany) and a Qubit Fluorometer (ThermoFisher Scientific, Waltham, MA, USA). cDNA synthesis was performed as described above. An overview of the respective techniques utilised for each sample is displayed in Supplementary Table S1.

The study was approved by the bioethics committee—ethical board approval number KB/430-74/20. This was retrospective non-interventional study and all the data analysed were collected as part of a routine clinical practice for diagnosis and treatment. All of the patients signed an informed consent form for treatment as per the standard operating procedures in our hospitals. In addition, the patients were diagnosed and treated following the national guidelines and policies. 

### 2.3. Nanopore and Sanger Sequencing 

Long-read sequencing (nanopore) was performed in three melanoma cell lines (NZM3, NZM6, and NZM15) and a non-malignant melanocyte cell line (Mel-ST). The complete *BARD1* sequence was amplified, as previously described [31]. Subsequent to gel electrophoresis (on 0.8% agarose gel), the PCR product was purified using Agencourt AMPure XP (Beckman Coulter, Brea, CA, USA). The libraries were prepared using the Direct cDNA Native Barcoding Kit (SQK-DCS109 with EXP-NBD104), and ~22 fmol of each library were pooled. To identify high-confidence *BARD1* isoforms, the FLAIR pipeline was used to align nanopore reads using splice aware options to human genome (hg19, www.gencodegene.org) (accessed on 24 December 2020). The --stringent option was used to return high-confidence isoforms that had at least three supporting reads (in at least one cell line) that spanned >80% of a given isoform with 25 bp in the 5′ and 3′ exons [32].

The presence of Δ(E3_E9, ▼(i8), IVS10▼176, and IVS10+131▼46 was validated using Sanger sequencing. All of the primers used for amplification and the subsequent Sanger sequencing are displayed in Table S2. 

### 2.4. RT-qPCR Data 

RT-qPCR was performed on 12 New Zealand melanoma cell lines and 18 archival melanoma FFPE tissues samples. To amplify the six previously reported *BARD1* isoforms, named β, δ, γ, ε, η, and φ, as well as four novel splice events (Δ(E3_E9), ▼(i8), IVS10▼176, and IVS10+131▼46) we used primers targeting specific exon–exon junctions (β, δ, γ, ε, η, φ, and Δ(E3_E9)) or, alternatively, exon–intron junctions ▼(i8), IVS10▼176 and IVS10+131▼46). The primer sequences used for RT-qPCR analysis are listed in Table S2. Real-time quantitative PCR was then performed on a LightCycler 480 instrument (Roche) using TB green Ex Taq master mix (TaKaRa, San Jose, CA, USA) and 5 nM of the appropriate primer pairs. All samples were performed in triplicate with amplification over 50 cycles, which were analysed using qbase+ software (version 2.7.11; http://www.biogazelle.com/qbaseplus (accessed on 24 December 2020); Biogazelle, Ghent, Belgium). To calculate the ΔCt values, the raw Ct values were normalised using TATA-binding protein (TBP), ubiquitin C (UBC), and phosphoglycerate kinase 1 (PGK1) as reference genes.

### 2.5. RNA-seq Data

RNA-Seq data generated from a sample of human melanocytes, two melanocyte cell lines (HEMnLP, HEMaLP), and three melanoma cell lines were used for the RNA-Seq analysis. The melanoma cell line data were generated from the same three cell lines as those used to generate the nanopore data (NZM 3, 6, and 15); however, the melanocyte data were from different cell lines. Reads were adaptor trimmed using cleanadaptors and aligned to the human reference genome GRCh37 using the alignment tool STAR [33]. Assembly was performed with the GENCODE v29 gene annotations and Stringtie [34]. The transcripts per kilobase million (TPM) expression counts for the annotated *BARD1* isoforms were obtained from the Stringtie gtf file. For the detection of novel isoforms, the BAM files generated by STAR were visualised in IGV to identify whether junction reads were detected to support novel splicing events and retained introns; however, this analysis was not quantitative. 

More information on the screening protocols can be obtained from the corresponding author upon request.

### 2.6. Sequencing Data Availability

Nanopore sequencing data supporting the conclusions of this manuscript will be made available by the authors, without undue reservation, to any qualified researcher upon request.

## 3. Results

### 3.1. Long-Range Nanopore Sequencing

**Figure 1:**
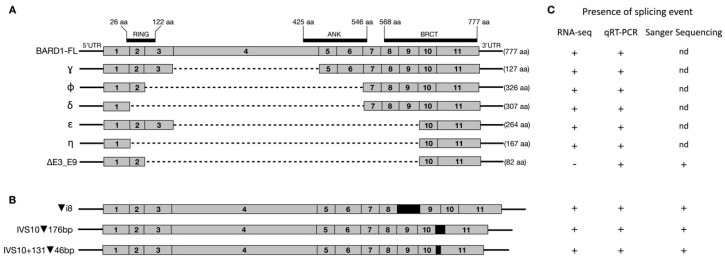
Schematic diagram of *BARD1* and identified isoforms (selected). (**A**) Structure of full-length *BARD1* (*BARD1*-FL) and six abundantly expressed alternative isoforms (γ, φ, δ, ε, η, and Δ(E3_E9)). (**B**) Structure of identified splicing events. (**C**) Presence of identified isoforms as detected using various sequencing techniques. (nd)- not done.

Herein, we present, for the first time, the expression of alternative *BARD1* splice isoforms in various melanoma samples. Using long-range PCR and nanopore sequencing, we identified a set of mRNAs corresponding to 76 different *BARD1* splice variants in total (Table S3), with six variant transcripts being expressed predominately (Figure 1A). Within these six, we identified a deletion encompassing exons 3 to 9 of *BARD1* (Δ(E3_E9); r.216_1903del), resulting in a frame shift, creating a premature stop codon (p.Glu646Ter). 

Moreover, nanopore sequencing analyses identified three different splicing events: (1) retention of intron 8 (▼(i8); r.1810_1811ins562), (2) inclusion of 175 bp from intron 10 (IVS10▼176; r.2001_2002ins2001+1_2001_176), and (3) activation of intronic exon within intron 10 (IVS10+131▼46; r.2001_2002ins2001+131_2001+176) (Figure 1B). Interestingly, all three splicing events were identified in several different transcripts, co-occurring with various other splicing arrangements, e.g., (▼(i8) was observed in four independent transcripts: row numbers 40, 44, 58, and 59. Similarly, IVS10▼176 and IVS10+131▼46 were present in 13 and six transcripts, respectively (Table S3). The presence of all of the above isoforms was confirmed with Sanger sequencing, which revealed characteristics of exon–exon or exon–intron junctions (Figure 1C and Figure S1). 

**Figure 2:**
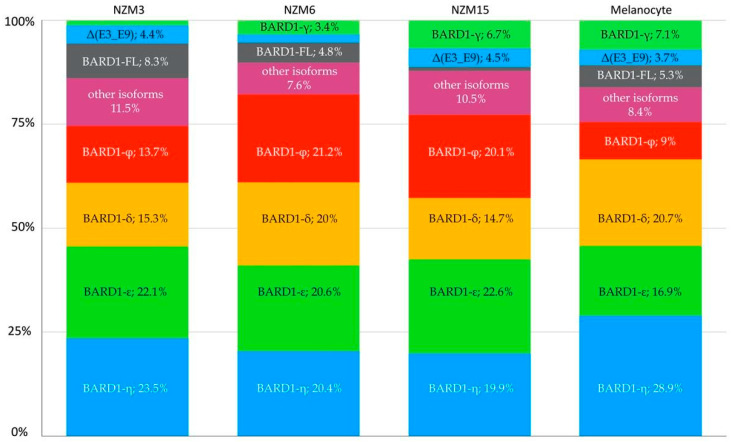
Proportion of *BARD1* transcript isoforms quantified using nanopore sequencing. The first three bars correspond to the New Zealand melanoma (NZM) cell lines, and the last bar corresponds to the non-malignant (melanocyte) control.

Among the identified transcripts, semi-quantitative analysis of the mean expression levels was highest for *BARD1-ε* and *BARD1-η* at 21.8% (range 20.6–22.6%) and 21.3% (range 19.9–23.5%), respectively (Figure 2). In non-malignant melanocytes, *ε* and *η* accounted for 16.9% and 28.9% of the expressed transcripts, respectively. *BARD1-δ* was more abundantly expressed in melanocytes, at 20.7%, than in NZM melanoma cell lines (mean expression 16.7%). *BARD1-φ* had a decreased representation within the melanocyte cell line (9% of total transcripts expressed), compared to the mean expression level of 18.3% (range 13.7–21.2%) in the NZM melanoma cell lines. Surprisingly, considering that amplification of full-length transcripts was efficiently achieved, the *BARD1*-FL was not the predominantly expressed transcript, contributing only 5.3% of total transcripts in melanocytes. Although, a possible PCR bias cannot be excluded via preferential amplification of shorter PCR products. Similarly, the mean expression was 4.7% (range 1–8.3%) in the NZM cell lines. The novel variant, Δ(E3_E9), was amongst the highest expressed transcripts present in both the melanoma and melanocyte cell lines with a frequency of 3.6% (range 2–4.5%) and 3.7%, respectively. 

### 3.2. RT-qPCR 

Next, by using RT-qPCR, we successfully validated the nanopore sequencing results and quantified all analysed transcripts, both in the cell lines and the archival (FFPE) melanoma samples (Figure S2. *BARD1-ɣ* was the highest expressed alternative isoform in the FFPE samples (mean normalised value 2.15), followed by *BARD1-η*, *BARD1-π*, and *BARD1-β*, accounting for, respectively, 1.72, 1.58, and 1.53. *BARD1-δ* was the lowest expressed transcript in the archival material (mean normalised value 0.27). In the NZM cell lines, *BARD1-β*, *BARD1-ɣ,* and *BARD1-η* were similarly expressed, with a mean normalised value of 1.41 to 1.43. In contrast to the nanopore sequencing results, *BARD1-δ* had the lowest expression level (0.135). In the non-malignant melanocyte control, the highest expression was observed for *BARD1-η* (3.00) and the lowest for *BARD1-δ* (0.005). Among the novel isoforms, *Δ(E3_E9)* was the highest expressed in the analysed FFPE samples (mean normalised value 0.597, range 0.05 to 0.75) (Figure S3). 

### 3.3. RNA-Seq

Lastly, to assess whether RNA-Seq was consistent with the other methods used, we analysed RNA-Seq datasets that were generated from the same melanoma cell lines used for nanopore sequencing and RT-qPCR. To enable quantification of the annotated *BARD1* isoform transcripts, the data were aligned in STAR and then assembly was performed using Stringtie. 

Interestingly, we observed that the proportion of each isoform transcript detected by Stringtie was not consistent with the nanopore sequencing data. The full-length *BARD1* isoform transcript was the most abundantly expressed, as reported by the software in all of the analysed RNA-Seq datasets (Table S4). As the RNA-Seq analysis only enabled detection of the annotated isoform mRNAs, we also visualised the BAM files in IGV to establish whether there were any reads to support novel splice variants. No junction reads were detected in any of the datasets to support the Δ(E3_E9) isoform. We did identify reads in the RNA-Seq data that supported the retention of intron 8 and 10; however, we were unable to assign these to a specific transcript.

## 4. Discussion

We carried out the first detailed analysis of alternative *BARD1* transcription in melanoma. To do this, we used a combination of nanopore sequencing, RNA sequencing, and RT-qPCR analyses on RNA from three melanoma cell lines and a melanocyte cell line. Nanopore sequencing enabled end-to-end sequencing across the entire length of PCR-amplified *BARD1* transcripts. Conversely, as RNA-Seq covers relatively short RNA fragments, only limited information can be derived about transcript structure. Moreover, using the nanopore sequencing of *BARD1* mRNA transcripts in melanoma, we identified the full-length *BARD1* isoform, along with five other major alternatively spliced transcripts. We additionally sequenced four novel *BARD1* splicing events in the melanoma cell lines, several of which we are reporting for the first time. 

Alternative *BARD1* transcripts contribute to the genesis of multiple *BARD1* isoforms, including α, β, κ, γ, φ, δ, ε, η, and ω. While multiple isoforms are expressed across various cancers, the relative expression levels of specific isoforms have been observed to vary [16]. Among these isoforms, the *BARD1-β* and *BARD1-δ* isoforms have attracted significant attention in cancer genomics due to their tumorigenic characteristics and ability to confer resistance to apoptosis [18]. In melanoma cell lines, however, nanopore sequencing identified the expression level of *BARD1-β* comprised <1% of total *BARD1* transcripts (Table S3). In contrast, the expression levels of the *BARD1-δ* transcript comprised between 14.7% and 20% of the *BARD1* transcripts observed. The upregulation of the *BARD1-δ* isoform expression in cancer has been associated with mitochondrial regulation in response to stress, but not with apoptosis stimulation or altering cell membrane permeability [35]. 

The other major isoform transcripts detected in melanoma using nanopore sequencing and RNA-Seq included *BARD1-γ*, *BARD1-φ*, *BARD1-ε,* and *BARD1-η*. However, the expression levels of the *BARD1*-FL transcripts were very low in all NZM melanoma cell lines, as measured by nanopore sequencing. Interestingly, the proportion of *BARD1*-FL expressed in our analyses was not concordant with the earlier study [31], despite using the same primer set, although we cannot explain this at present. Structural analyses of the *BARD1* isoforms *δ*, *ε,* and *η* predict an absence of the ANK-BRCT binding domain, or part of the BRCT region. These motifs are crucial sites for binding with several tumour suppressor proteins, including TP53 [36,37,38]. *BARD1*–TP53 complex formation is fundamental for stimulating nuclear export and mitochondrial localisation, and is subsequently required to induce apoptosis [39]. The induction of TP53-mediated apoptosis via *BARD1* occurs irrespective of the serine-15 phosphorylation status of TP53 [36,40]. Moreover, *BARD1*-δ antagonises *BARD1*-FL. As such, increased levels of *BARD1*-δ expression relative to *BARD1*-FL may regulate or inhibit tumour suppressor functions in melanoma cell lines. 

Reduced expression of *BARD1* isoforms containing the functional RING domain may result in decreased BRCA1–*BARD1* interactions [41]. Dimerisation of BRCA1 and *BARD1* is required to stimulate their shared ubiquitination activity and is required for the heterodimer tumour suppressor functions [42]. Taken together, the relatively high expression of *BARD1-δ*, *BARD1-ε,* and *BARD1-η*, compared to *BARD1*-FL in melanoma cells, suggests a role for these transcripts in uncontrolled melanoma cell proliferation and mitochondrial regulation in response to stress. However, despite support for a pro-tumour role of these isoforms in the literature, significant differences were not observed between our melanocyte and melanoma data; thus, further investigations are warranted.

Nanopore and Sanger sequencing has identified two other *BARD1* splice variants of interest in melanoma: *BARD1 Δ(E3_E9)* and *IVS10+131▼46* (Figure 1). These splicing events have previously been identified in lymphoblastoid cell lines [31]. Splice variant *Δ(E3_E9)* was also detected by RT-qPCR in the FFPE melanoma samples, but was unable to be detected in the RNA-Seq data. There are inherent limitations to the use of RNA-Seq for the identification of novel splice variants. This is primarily due to the reliance of assembly tools on annotations for accurate transcript assembly. As the novel *Δ(E3_E9)* splice variant is not within the Gencode annotations, Stringtie was unable to detect this isoform. We attempted to identify junction reads between exons 3 and 9 using IGV. However, none were detected using this approach and thus we were unable to confirm the presence of this splicing event within the RNA-Seq data. This emphasises a significant limitation of RNA-Seq data in the detection of splice variants. The relatively short reads generated by RNA-Seq experiments make assignment to specific transcripts difficult, particularly when dealing with a gene that shows complex splicing, such as *BARD1*. Additionally, this may have led to an overestimation of the full-length isoform expression in the RNA-Seq data when compared to nanopore sequencing (Tables S3 and S4). Stringtie takes reads that have aligned to *BARD1* and then performs transcript assembly, generating a count for each annotated isoform. Therefore, reads derived from the novel splice isoforms detected by nanopore sequencing may have been assigned to the full-length *BARD1* by Stringtie, due to the lack of annotations for these transcripts. Similarly, this may be used to explain the non-concordance of the results observed for other well documented isoforms, e.g., Gamma (Δ(E4)), as this splice event is present in multiple independent transcripts. Unfortunately, we were unable to validate this possible occurrence in the RNA-Seq data via RT-qPCR, as there are no unique sequences within the *BARD1*-FL isoform such that it could be confidently differentiated from other annotated isoforms. 

The role of these isoforms in human biology is largely unknown. In previous studies, the roles of *Δ(E3_E6)* and *Δ(E3_E7)* in cell proliferation have been described, with the ΔE3 isoform using an alternative open reading frame to encode functional proteins [43]. In contrast to Walker et al., [31], the *IVS10+131▼46* was also present in several different transcripts (co-occurring with other splicing alterations). This might suggest its potential importance in melanoma biology. Similarly, the novel splicing events, named *▼(i8)* and *IVS10▼176* (which comprise less than 1% of *BARD1* transcripts), were also noted in several different transcripts. A frame shift in the *BARD1* open reading frame (ORF) resulted both from insertion of intron 8 sequences for the *▼(i8)* splice event and the inclusion of 175 bp from intron 10 in the *IVS10▼176* splice event. Such frame shift indels could lead to mRNA degradation, owing to nonsense-mediated mRNA decay or the production of truncated proteins [44]. Further work is required to investigate the functional significance of these variants.

## 5. Conclusions

In conclusion, we identified multiple alternative transcripts corresponding to known *BARD1* isoforms, expressed at much higher levels than *BARD1*-FL in both melanoma and melanocytes. While these results observed in melanoma could be expected from the literature, the same observation in the control melanocytes was unexpected. The identified variants encode proteins lacking the domains required for BRCA1 interaction and tumour suppressor functions. These results therefore do not support a pro-tumorigenic role of *BARD1* isoforms in melanoma, although further investigation will be required. Important limitations of the current study include the degree of variation observed between the different sequencing methods. The apparent lack of expression of *BARD1* isoforms using RNA-Seq may be attributed to short reads being falsely assigned to *BARD1*-FL. Nevertheless, using nanopore sequencing technology, we unexpectedly found that what are usually rare and potentially antagonistic isoforms of *BARD1* transcription are in fact expressed at relatively high levels in melanoma and melanocytes.

## Data Availability

All data presented in this study, including nanopore sequencing data, is available upon request to the corresponding author.

## Supplementary Materials

The following are available online at https://www.mdpi.com/2073-4425/12/2/320/s1, Figure S1: Validation of Nanopore results by Sanger sequencing, Figure S2: Relative expression of predominant BARD1 isoforms in melanoma tissues, melanoma cell lines and melanocytes, Figure S3: Relative expression of novel BARD1 splice events in melanoma tissues, melanoma cell lines and melanocytes, Table S1: An evaluation of samples included in each technique, Table S2: Primer sequences used in the study, Table S3: BARD1 transcript variants identified using long-range Nanopore sequencing, Table S4: Expression of annotated BARD1 transcripts from RNA-Seq data.
