# Supplementary material for: Differential Expression of BARD1 Isoforms in Melanoma
Source: Genes (Basel). 2021 Feb 23;12(2):320. doi: 10.3390/genes12020320 (PMC7927127; doi:10.3390/genes12020320)
Supplement: Supplementary file 1 [file genes-12-00320-s001.zip › 20210211 Supplementary materials_RP.pdf]

genes MDPI

Table S1. An evaluation of samples included in each technique

Sample	Nanopore	Sanger Sequencing	RNAseq	RT-qPCR
FFPE patient tissue	X	X	X	✓
NZM3	✓	✓	✓	✓
NZM6	✓	✓	✓	✓
NZM11	X	X	✓	✓
NZM12	X	X	✓	✓
NZM15	✓	✓	✓	✓
NZM18	X	X	X	✓
NZM20	X	X	✓	✓
NZM22	X	X	✓	✓
NZM25	X	X	X	✓
NZM40	X	X	✓	✓
NZM45	X	X	✓	✓
NZM53	X	X	✓	✓
Melanocyte	✓	✓	✓	✓

Table S2. Primer sequences used in the study

Primer name	Forward primer sequence	Reverse Primer sequence
**RT-qPCR**		
BARD1-β	CTGCTCGCGTTGATTTGAAAG	ACTTCGAGGGCTAAACCACA
BARD1-δ	TGCTCGCGTTGTAATATATTTG	GCTGCCCAGTGTTCATTACTG*
BARD1-γ	CAATGAGCTGTCAGGGCGAC	GCAAGCTTCATGCAATGGTG
BARD1-ε	TGAGCTGTCAGGGGTAAAAG	CAAACAGCTTTGGCAACAGCTG#
BARD1-η	TGCTCGCGTTGGGGTAAAAG	CAAACAGCTTTGGCAACAGCTG#
BARD1-φ	CTGTAGTAATATATTTGGTCTGC	GCTGCCCAGTGTTCATTACTG*
BARD1-Δ(E3_E9)	CATCTTCTGTAGGGGTAAAAG	CTCTGTTGAGCCTGCTTCTG
BARD1-▼(i8)	GAGTTTGACAGTACAGGTGAG	GGTAGTTCTCCAAAAGGATCA
BARD1-IVS10▼176	GGTAAAAGCATGTCTACGAA	CAAACAGCTTTGGCAACTTCTG
BARD1-IVS10+131▼46	AACAGAGAACAGCTGCACTTG	CTGGCTTGGGCTTTCTACTG
TBP	GAACATCATGGATCAGAACAACA	ATAGGGATTCCGGGAGTCAT
UCB	GCAAAGATCCAAGATAAGGAA	GGACCAAGTGCAGAGTGGAC
PGK1	TGCAAAGGCCTTGGAGAG	TGGATCTTGTCTGCAACTTTAGC
**Sanger Sequencing**		
BARD1-Δ(E3_E9)	GCACATCTTCTGTAGGGGTA	CTCTGTTGAGCCTGCTTCTG
BARD1-▼(i8)	GAGTTTGACAGTACAGGTGAG	TTGAACTGCATCACCAGGAA
BARD1-IVS10▼176	ATGCTTGGGATTCTCAATGG	CAAACAGCTTTGGCAACTTCTG
BARD1-IVS10+131▼46	AAC AGA GAA CAG CTG CAC TTG	CTGGCTTGGGCTTTCTACTG

Table S3. BARD1 transcript variants identified using long-range Nanopore sequencing

No.	Splicing event description	RNA change	Number of reads in Nanopore			
			NZM 3	NZM 6	NZM 15	MelSt

1 **Formatted:** Indent: Left: 0 cm

2 **Formatted:** Indent: Left: 0 cm

Deleted: A1

Deleted: analysis

Commented [MR1]: If it failed (qPCR) than probably we should remove this one.

3 **Deleted:** A2

1	Full length	none	5193	2982	667	3309
2	BARD1-β; Δ(E2_E3)	r.159_364del	619	72	102	144
3	BARD1-δ; Δ(E2_E6)	r.159_1568del	9537	10288	5495	10087
4	BARD1-η; Δ(E2_E9)	r.159_1903del	14671	10522	7387	14052
5	BARD1-κ; Δ(E3)	r.216_364del	950	349	288	337
6	BARD1- γ; Δ(E4)	r.365_1314del	797	917.0	1126	2160
7	BARD1-π; Δ(E4q)	r.907_1314del	187	161	6	11
8	BARD1-ε; Δ(E4_E9)	r.365_1903del	13794	10588	8425	8206
9	BARD1- φ; Δ(E3_E6)	r.216_1568del	8543	10924	7488	4372
10	Δ(E2_E3,E4q)*	r.159_364del+907_1314del	50	1	3	25
11	Δ(E2_E3,E5_E9)*	r.159_364del+1315_1903del	86	51	42	64
12	Δ(E2,E4)	r.159_1314del	12	1	8	50
13	Δ(E2,E4_E9)	r.159_215+365_1903del	129	117	45	87
14	Δ(E2_E4)	r.159_1314del	99	170	386	221
15	Δ(E2_E4,E7_E9)*	r.159_1314del+1569_1903del	27	21	14	134
16	Δ(E2_E4,E8)	r.159_1314del+1678_1810del	7	1	0	2
17	Δ(E2_E6)	r.159_1568del	6	3	6	7
18	Δ(E2_E6,E8)	r.159_1568+1678_1810del	95	1	3	2
19	Δ(E2_E6,E8_E9)	r.159_1568+1678_1903del	28	21	16	19
20	Δ(E2_E6,E9_E10)*	r.159_1568+1811_2001del	2	2	2	2
21	Δ(E2_E6,E10)*	r.159_1568+1904_2001del	4	0	0	0
22	Δ(E2_E6),IVS10▼176*	r.159_1568+2001_2002ins2001+1_2001_176	26	13	4	19
23	Δ(E2_E6),IVS10+131▼46*	r.159_1568+2001_2002ins2001+131_2001+176	78	106	45	79
24	Δ(E2_E7)	r.159_1677del	215	52	179	168
25	Δ(E2_E7,E9)	r.159_1677del+ 1811_1903del	81	57	28	66
26	Δ(E2_E7),IVS10▼176*	r.159_1677del+2001_2002ins2001+1_2001_176	5	0	1	3
27	Δ(E2_E7),IVS10+131▼46*	r.159_1677del+2001_2002ins2001+131_2001+176	5	1	3	4
28	Δ(E2_E8)*	r.159_1810del	9	1	4	1
29	Δ(E2_E9),IVS10▼176*	r.159_1903del+2001_2002ins2001+1_2001_176	302	3	2	238
30	Δ(E2_E10),IVS10+131▼46*	r.159_2001del+2001_2002ins2001+131_2001+176	233	79	47	112
31	Δ(E3,E5)*	r.216_1395del+1315_1395del	5	3	7	12
32	Δ(E3,E5_E6)*	r.216_364del+1315_1568del	113	115	56	76
33	Δ(E3,E5_E9)	r.216_364del+1315_1903del	89	74	43	59
34	Δ(E3),IVS10▼176*	r.216_364del+2001_2002ins2001+1_2001_176	42	2	1	20
35	Δ(E3),IVS10+131▼46*	r.216_364del+2001_2002ins2001+131_2001+176	20	2	0	5
36	Δ(E3_E4)	r.216_1314del	380	171	714	293
37	Δ(E3,E4q)	r.216_364del+907_1314del	66	4	2	4
38	Δ(E3_E4,E6)*	r.216_1314del+1396_1568del	180	197	330	197
39	Δ(E3_E4,E8)	r.216_1568+1678_1810del	49	0	0	4
40	Δ(E3_E4),▼(i8)*	r.216_1314del+1810_1811ins562	14	46	9	4
41	Δ(E3_E4),IVS10▼176*	r.216_1314del+2001_2002ins2001+1_2001_176	41	0	87	5

Formatted ... [1]
Formatted ... [2]
Formatted ... [3]
Formatted ... [4]
Formatted ... [5]
Formatted ... [6]
Formatted ... [7]
Formatted ... [8]
Formatted ... [9]
Formatted ... [10]
Formatted ... [11]
Formatted ... [12]
Formatted ... [13]
Formatted ... [14]
Formatted ... [15]
Formatted ... [16]
Formatted ... [17]
Formatted ... [18]
Formatted ... [19]
Formatted ... [20]
Formatted ... [21]
Formatted ... [22]
Formatted ... [23]
Formatted ... [24]
Formatted ... [25]
Formatted ... [26]
Formatted ... [27]
Formatted ... [28]
Formatted ... [29]
Formatted ... [30]
Formatted ... [31]
Formatted ... [32]
Formatted ... [33]
Formatted ... [34]
Formatted ... [35]
Formatted ... [36]
Formatted ... [37]
Formatted ... [38]
Formatted ... [39]
Formatted ... [40]
Formatted ... [41]
Formatted ... [42]
Formatted ... [43]
Formatted ... [44]
Deleted: 2

42	Δ(E3_E5)	r.216_1395del	21	2	2	7
43	Δ(E3_E6,E8)	r.216_1568+1678_1810del	92	3	6	4
44◎	Δ(E3_E6),▼(i8)*	r.216_1568+1810_1811ins562	20	15	4	22
45	Δ(E3_E6,E8_E10)*	r.216_1568+1678_2001del	1	1	3	2
46	Δ(E3_E6,E9)*	r.216_1568+1811_1903del	90	59	36	65
47⊠	Δ(E3_E6),IVS10▼176*	r.216_1568+2001_2002ins2001+1_2001_176	20	6	6	7
48□	Δ(E3_E6),IVS10+131▼46*	r.216_1568+2001_2002ins2001+131_2001+176	118	87	14	14
49	Δ(E3,E7)	r.216_364del+1569_1677del	6	0	2	4
50	Δ(E3_E7)	r.216_1677del	683	768	130	647
51	Δ(E3,E7_E9)	r.216_364del+ 1569_1903del	42	16	15	28
52⊠	Δ(E3_E7),IVS10▼176*	r.216_1677del+2001_2002ins2001+1_2001_176	68	0	0	3
53	Δ(E3,E8)	r.216_364del+ 1678_1810del	11	0	4	2
54	Δ(E3_E8)	r.216_1810del	95	185	3	8
55◉	Δ(E3_E9)	r.216_1903del	2759	1335	2779	2312
56	Δ(E4,E7)	r.365_1314del+1569_1677del	19	102	7	8
57	Δ(E4,E8_E9)	r.365_1314del+1678_1903del	25	23	54	28
58◎	Δ(E4),▼(i8)*	r.365_1314del+1810_1811ins562	20	15	4	22
59⊠◎	Δ(E4),▼(i8), IVS10▼176*	r.365_1314del+1810_1811ins562+2001_2002ins2001+1_2001_176	9	1	0	2
60⊠	Δ(E4),IVS10▼176*	r.365_1314del+2001_2002ins2001+1_2001_176	72	3	7	71
61⊠	Δ(E4q),IVS10▼176*	r.907_1314del+2001_2002ins2001+1_2001_176	9	0	2	4
62	Δ(E4_E6)	r.365_1568del	597	343	832	81
63	Δ(E4_E6,E8)	r.365_1568del+1678_1810del	10	3	3	42
64⊠	Δ(E4_E6),IVS10▼176*	r.365_1568del+1810_1811ins562+2001_2002ins2001+1_2001_176	11	1	1	2
65	Δ(E4_E7)	r.365_1677del	187	6	13	69
66	Δ(E4_E8)	r.365_1810del	112	3	1	8
67⊠	Δ(E4_E9),IVS10▼176*	r.365_1903del+2001_2002ins2001+1_2001_176	32	13	8	15
68□	Δ(E4_E9),IVS10+131▼46*	r.365_1903del+2001_2002ins2001+131_2001+176	36	18	17	27
69	Δ(E5)	r.1315_1395del	19	3	3	11
70	Δ(E5_E9)	r.1315_1903del	93	72	58	63
71	Δ(E6)	r.1396_1568del	210	193	94	173
72	Δ(E7)	r.1569_1677del	27	14	16	16
73	Δ(E8_E9)	r.1678_1903del	33	32	12	35
74	Δ(E9_E10)	r.1811_2001del	6	3	1	5
75⊠	IVS10▼176*	r.2001_2002ins2001+1_2001_176	70	2	10	13
76□	IVS10+131▼46	r. 2001_2002ins2001+131_2001+176	22	3	2	25

* indicates novel isoforms identified in this study (not reported by Walker et al., 2019).
⊠ indicates all transcripts with retention of 176 bp in intron 10 (IVS10▼176).
□ indicates all transcripts with activation of 46 bp intronic-exon (IVS10+131▼46).
◎ indicates all trnascripts with retention of intron 8 (▼(i8)).
◉ indicates transcripts with exon 3 to 9 dleletion (Δ(E3_E9)).

Deleted: 3
Formatted ... [45]
Deleted: 4
Formatted ... [46]
Deleted: 5
Formatted ... [47]
Deleted: 6
Formatted ... [48]
Formatted ... [49]
Deleted: 7
Formatted ... [50]
Deleted: 8
Formatted ... [51]
Deleted: 9
Deleted: 50
Formatted ... [52]
Deleted: 4
Formatted ... [53]
Deleted: 1
Formatted ... [54]
Formatted ... [55]
Formatted ... [56]
Formatted ... [57]
Formatted ... [58]
Deleted: *
Formatted ... [59]
Formatted ... [60]
Formatted ... [61]
Formatted ... [62]
Formatted ... [63]
Formatted ... [64]
Formatted ... [65]
Formatted ... [66]
Formatted ... [67]
Formatted ... [68]
Formatted ... [69]
Formatted ... [70]
Formatted ... [71]
Formatted ... [72]
Formatted ... [73]
Formatted ... [74]
Formatted ... [75]
Formatted ... [76]
Formatted ... [77]
Formatted ... [78]
Formatted ... [79]
Formatted Table ... [80]
Formatted ... [81]
Formatted ... [82]
Formatted ... [83]
Formatted ... [84]
Formatted ... [85]
Formatted ... [86]

Table S4. Expression of annotated BARD1 transcripts from RNA-seq data.

Transcript name	Ensembl ID	Transcripts per million killobases (TPM)												
		Melanoma cell lines										Melanocyte cell lines		
		NZM3	NZM6	NZM 11	NZM 12	NZM 15	NZM 20	NZM 22	NZM 40	NZM 45	NZM 50	M 1	M 2	M 3
FL-BARD1	ENST00000260947.9_2	4.31	3.43	6.851	8.085	8.35	2.64	9.22	5.95	11.614	3.174	4.94	4.30	3.94
BARD1-204 (NMD)	ENST00000455743.5_1	1.91	1.03	3.714	0.416	3.50	0.00	0.00	0.659	0.634	1.931	2.32	1.22	0.56
BARD1- γ; Δ(E4)	ENST00000620057.4_1	0.25	1.03	1.526	0.538	0.63	0.348	1.14	0.503	2.992	0.670	0.00	0.00	0.00
BARD1-208 (NC, retained intron)	ENST00000479904.1_1	0.22	0.29	0.240	0.139	0.33	0.057	0.09	0.175	0.306	0.173	0.05	0.00	0.00
BARD1-215 (NMD; starts in ex 2)	ENST00000650978.1_1	0.11	0.07	1.168	0.780	0.94	0.020	0.283	0.075	0.846	0.272	0.28	0.02	0.07
BARD1-206 (NC)	ENST00000471590.5_1	0.05	0.00	0.051	0.156	0.07	0.01	0.06	0.125	0.029	0.023	0.01	0.00	0.04
BARD1-203 (ex10/11 only)	ENST00000432456.5_2	0.03	0.00	0.033	0.024	0.00	0.00	0.022	0.032	0.00	0.019	0.04	0.11	0.01
BARD1-205 (NC, retained intron)	ENST00000465841.1_1	0.00	0.00	0.016	0.022	0.02	0.013	0.00	0.00	0.021	0.018	0.01	0.01	0.02
BARD1-ε; Δ(E4_E9)	ENST00000619009.5_2	0.00	0.00	0.00	0.00	0.00	0.00	0.00	0.00	0.186	0.00	0.00	0.00	0.00
BARD1-α; Δ(E2)	ENST00000617164.5_2	0.00	0.00	0.00	0.00	0.00	0.00	0.00	0.00	0.517	0.00	0.00	0.00	0.00
BARD1-δ; Δ(E2_E6)	ENST00000613374.5_2	0.00	0.00	0.00	0.00	0.00	0.00	0.00	0.00	0.00	0.112	0.00	0.00	0.00
BARD1- φ; Δ(E3_E6)	ENST00000421162.2_3	0.00	0.00	0.00	0.00	0.00	0.00	0.00	0.00	0.00	0.00	0.00	0.00	0.00
BARD1-π; Δ(E4q)	ENST00000613706.5_2	0.00	0.11	0.00	0.00	0.00	0.00	0.00	0.00	0.00	0.278	0.00	0.00	0.00
BARD1-209 (NMD)	ENST00000613192.2_2	0.00	0.00	0.00	0.00	0.00	0.00	0.00	0.00	0.00	0.00	0.00	0.00	0.00
BARD1-207 (NC)	ENST00000471787.1_1	0.00	0.07	0.00	0.00	0.00	0.00	0.00	0.00	0.00	0.00	0.09	0.00	0.00

NMD – Designates transcripts which undergo nonsense-mediated mRNA decay.
NC – Designates transcripts which do not encode a functional polypeptide.
Numerical symbols (E.g. BARD1-204) correspond to Ensembl transcript.

Formatted ... [87]
Deleted: A3
Formatted Table ... [88]
Formatted ... [89]
Formatted ... [90]
Deleted: NMD
Formatted ... [91]
Deleted: Gamma
Formatted ... [92]
Formatted ... [93]
Formatted ... [94]
Formatted ... [95]
Formatted ... [96]
Formatted ... [97]
Formatted ... [98]
Formatted ... [99]
Formatted ... [101]
Formatted ... [102]
Formatted ... [103]
Formatted ... [104]
Formatted ... [105]
Formatted ... [106]
Deleted: (
Formatted ... [100]
Formatted ... [107]
Formatted ... [108]
Formatted ... [109]
Formatted ... [110]
Formatted ... [111]
Formatted ... [112]
Formatted ... [114]
Formatted ... [115]
Formatted ... [116]
Formatted ... [113]
Formatted ... [117]
Formatted ... [118]
Formatted ... [119]
Formatted ... [120]
Formatted ... [121]
Formatted ... [122]
Formatted ... [123]
Deleted: 1-3/10-11 epsilon
Formatted ... [124]
Deleted: Alpha
Formatted ... [125]
Deleted: Delta
Formatted ... [126]
Deleted: Phi
Formatted ... [127]
Deleted: Pi
Formatted ... [129]
Formatted ... [128]
Formatted ... [132]
Formatted ... [133]

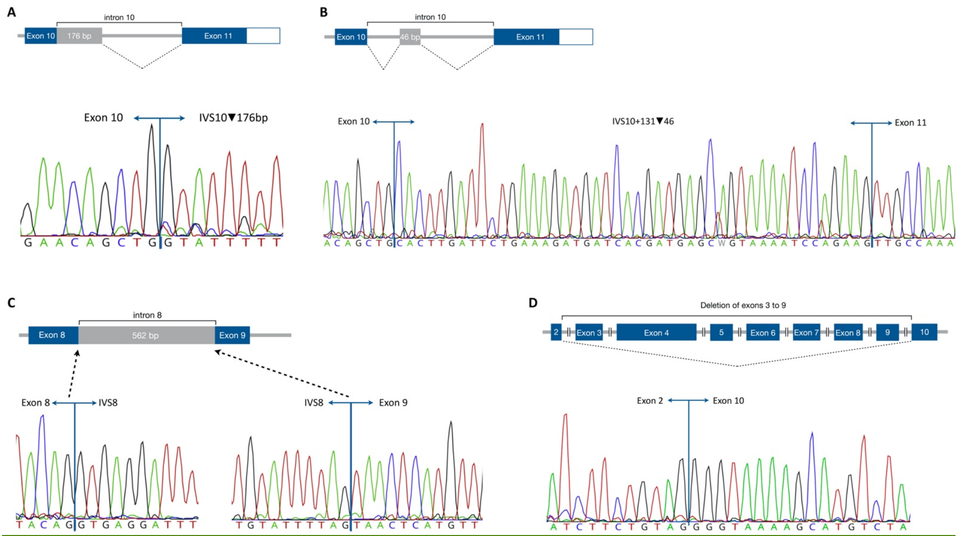

Figure S1. Validation of Nanopore results by Sanger sequencing. Top panels entail schematics corresponding to electropherogram for regions of interest below. A) Retention of 176 bp in intron 10. B) Activation of intronic exon within intron 10. C) Retention of intron 8 (left panel demonstrates exon 8-intron 8 junction, right panel demonstrates intron 8-exon 9 junction). D) Skipping of exons 3 to 9.

Formatted: Font: Palatino Linotype, Bold, Not Italic

Formatted: Caption

Formatted: Font: Palatino Linotype, Not Italic

Formatted: Font: Palatino Linotype, Not Italic

Formatted: Font: Palatino Linotype, Not Italic

Deleted: Appendix B

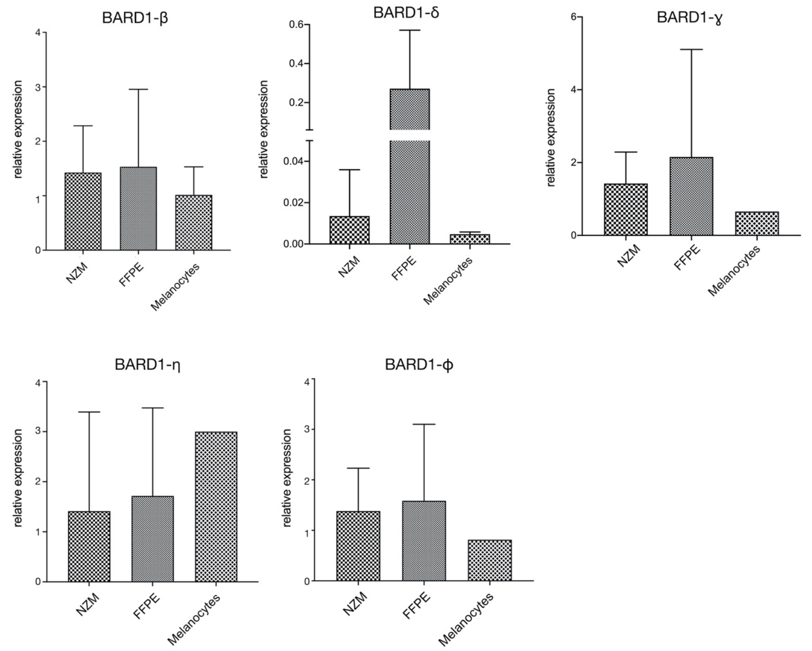

Figure S2. Relative expression of predominant BARD1 isoforms in melanoma tissues, melanoma cell lines and melanocytes. This was analysed in FFPE patient melanoma samples and melanoma cell lines (NZM3, NZM6, NZM15, NZM11, NZM12, NZM18, NZM20, NZM22, NZM25, NZM40, NZM45, NZM53) and melanocytes using RT-qPCR. Expression was normalised to reference genes TBP, UBC and PGK1. Amplification of BARD1-ε in FFPE was unsuccessful, therefore results are not presented.

Deleted: 1

Deleted: q

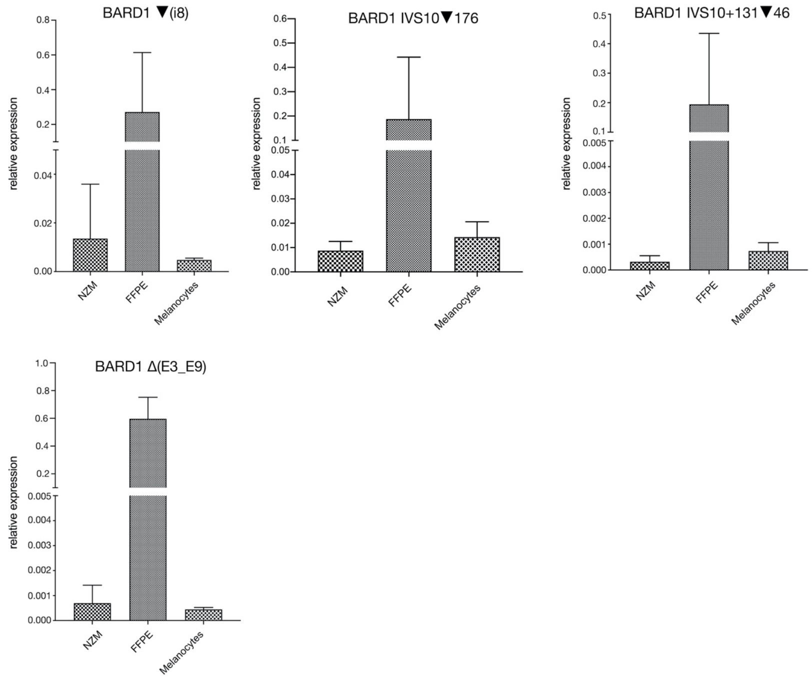

Figure S3. Relative expression of novel BARD1 splice events in melanoma tissues, melanoma cell lines and melanocytes. This was analysed in FFPE patient melanoma samples and melanoma cell lines (NZM3, NZM6, NZM15, NZM11, NZM12, NZM18, NZM20, NZM22, NZM25, NZM40, NZM45, NZM53) and melanocytes using RT-qPCR. Expression was normalised to reference genes TBP, UBC and PGK1.

Deleted: B

Deleted: 2

Deleted: q

Deleted: References

Formatted: Line spacing: single, Don't adjust space between Latin and Asian text, Don't adjust space between Asian text and numbers

Page 2: [1] Formatted	Ryan Powell	11/02/2021 11:29:00

Left

Page 2: [2] Formatted	Ryan Powell	11/02/2021 11:29:00

Left

Page 2: [3] Formatted	Ryan Powell	11/02/2021 11:29:00

Left

Page 2: [4] Formatted	Ryan Powell	11/02/2021 11:29:00

Left

Page 2: [5] Formatted	Ryan Powell	11/02/2021 11:29:00

Left

Page 2: [6] Formatted	Ryan Powell	11/02/2021 11:29:00

Left

Page 2: [7] Formatted	Ryan Powell	11/02/2021 11:29:00

Left

Page 2: [8] Formatted	Ryan Powell	11/02/2021 11:29:00

Left

Page 2: [9] Formatted	Ryan Powell	11/02/2021 11:29:00

Left

Page 2: [10] Formatted	Ryan Powell	11/02/2021 11:29:00

Left

Page 2: [11] Formatted	Ryan Powell	11/02/2021 11:29:00

Left

Page 2: [12] Formatted	Ryan Powell	11/02/2021 11:29:00

Left

Page 2: [13] Formatted	Ryan Powell	11/02/2021 11:29:00

Left

Page 2: [14] Formatted	Ryan Powell	11/02/2021 11:29:00

Left

Page 2: [15] Formatted	Ryan Powell	11/02/2021 11:29:00

Left

Page 2: [16] Formatted	Ryan Powell	11/02/2021 11:29:00

Left

Page 2: [17] Formatted **Ryan Powell** **11/02/2021 11:29:00**

Left

Page 2: [18] Formatted **Ryan Powell** **11/02/2021 11:29:00**

Left

Page 2: [19] Formatted **Ryan Powell** **11/02/2021 11:29:00**

Left

Page 2: [20] Formatted **Ryan Powell** **11/02/2021 11:29:00**

Left

Page 2: [21] Formatted **Ryan Powell** **11/02/2021 11:29:00**

Left

Page 2: [22] Formatted **Ryan Powell** **11/02/2021 11:29:00**

Left

Page 2: [23] Formatted **Ryan Powell** **11/02/2021 11:28:00**

Font: (Default) Segoe UI Symbol

Page 2: [24] Formatted **Ryan Powell** **11/02/2021 11:23:00**

Superscript

Page 2: [25] Formatted **Ryan Powell** **11/02/2021 11:32:00**

Superscript

Page 2: [26] Formatted **Ryan Powell** **11/02/2021 11:29:00**

Left

Page 2: [27] Formatted **Ryan Powell** **11/02/2021 11:29:00**

Left

Page 2: [28] Formatted **Ryan Powell** **11/02/2021 11:29:00**

Left

Page 2: [29] Formatted **Ryan Powell** **11/02/2021 11:29:00**

Left

Page 2: [30] Formatted **Ryan Powell** **11/02/2021 11:29:00**

Left

Page 2: [31] Formatted **Ryan Powell** **11/02/2021 11:29:00**

Left

Page 2: [32] Formatted **Ryan Powell** **11/02/2021 11:29:00**

Left

Page 2: [33] Formatted **Ryan Powell** **11/02/2021 11:29:00**

Left

Page 2: [34] Formatted **Ryan Powell** **11/02/2021 11:29:00**

Left

Page 2: [35] Formatted **Ryan Powell** **11/02/2021 11:29:00**

Left

Page 2: [36] Formatted **Ryan Powell** **11/02/2021 11:29:00**

Left

Page 2: [37] Formatted **Ryan Powell** **11/02/2021 11:29:00**

Left

Page 2: [38] Formatted **Ryan Powell** **11/02/2021 11:29:00**

Left

Page 2: [39] Formatted **Ryan Powell** **11/02/2021 11:29:00**

Left

Page 2: [40] Formatted **Ryan Powell** **11/02/2021 11:29:00**

Left

Page 2: [41] Formatted **Ryan Powell** **11/02/2021 11:29:00**

Left

Page 2: [42] Formatted **Ryan Powell** **11/02/2021 11:29:00**

Left

Page 2: [43] Formatted **Ryan Powell** **11/02/2021 11:29:00**

Left

Page 2: [44] Formatted **Ryan Powell** **11/02/2021 11:29:00**

Left

Page 3: [45] Formatted **Ryan Powell** **11/02/2021 11:29:00**

Left

Page 3: [46] Formatted **Ryan Powell** **11/02/2021 11:29:00**

Left

Page 3: [47] Formatted **Ryan Powell** **11/02/2021 11:29:00**

Left

Page 3: [48] Formatted	Ryan Powell	11/02/2021 11:29:00

Left

Page 3: [49] Formatted	Ryan Powell	11/02/2021 11:29:00

Left

Page 3: [50] Formatted	Ryan Powell	11/02/2021 11:29:00

Left

Page 3: [51] Formatted	Ryan Powell	11/02/2021 11:29:00

Left

Page 3: [52] Formatted	Ryan Powell	11/02/2021 11:29:00

Left

Page 3: [53] Formatted	Ryan Powell	11/02/2021 11:29:00

Left

Page 3: [54] Formatted	Ryan Powell	11/02/2021 11:29:00

Left

Page 3: [55] Formatted	Ryan Powell	11/02/2021 11:29:00

Left

Page 3: [56] Formatted	Ryan Powell	11/02/2021 11:29:00

Left

Page 3: [57] Formatted	Ryan Powell	11/02/2021 11:29:00

Left

Page 3: [58] Formatted	Ryan Powell	11/02/2021 11:29:00

Left

Page 3: [59] Formatted	Ryan Powell	11/02/2021 11:29:00

Left

Page 3: [60] Formatted	Ryan Powell	11/02/2021 11:29:00

Left

Page 3: [61] Formatted	Ryan Powell	11/02/2021 11:29:00

Left

Page 3: [62] Formatted	Ryan Powell	11/02/2021 11:29:00

Left

Page 3: [63] Formatted	Ryan Powell	11/02/2021 11:29:00

Left

Page 3: [64] Formatted	Ryan Powell	11/02/2021 11:29:00

Left

Page 3: [65] Formatted	Ryan Powell	11/02/2021 11:29:00

Left

Page 3: [66] Formatted	Ryan Powell	11/02/2021 11:29:00

Left

Page 3: [67] Formatted	Ryan Powell	11/02/2021 11:29:00

Left

Page 3: [68] Formatted	Ryan Powell	11/02/2021 11:29:00

Left

Page 3: [69] Formatted	Ryan Powell	11/02/2021 11:29:00

Left

Page 3: [70] Formatted	Ryan Powell	11/02/2021 11:29:00

Left

Page 3: [71] Formatted	Ryan Powell	11/02/2021 11:29:00

Left

Page 3: [72] Formatted	Ryan Powell	11/02/2021 11:29:00

Left

Page 3: [73] Formatted	Ryan Powell	11/02/2021 11:29:00

Left

Page 3: [74] Formatted	Ryan Powell	11/02/2021 11:29:00

Left

Page 3: [75] Formatted	Ryan Powell	11/02/2021 11:29:00

Left

Page 3: [76] Formatted	Ryan Powell	11/02/2021 11:29:00

Left

Page 3: [77] Formatted	Ryan Powell	11/02/2021 11:29:00

Left

Page 3: [78] Formatted	Ryan Powell	11/02/2021 11:29:00

Left

Page 3: [79] Formatted	Ryan Powell	11/02/2021 11:29:00

Left

Page 3: [80] Formatted Table **Ryan Powell** **11/02/2021 11:20:00**

Formatted Table

Page 3: [81] Formatted **Ryan Powell** **11/02/2021 11:33:00**

Superscript

Page 3: [81] Formatted **Ryan Powell** **11/02/2021 11:33:00**

Superscript

Page 3: [81] Formatted **Ryan Powell** **11/02/2021 11:33:00**

Superscript

Page 3: [81] Formatted **Ryan Powell** **11/02/2021 11:33:00**

Superscript

Page 3: [82] Formatted **Ryan Powell** **11/02/2021 11:37:00**

Superscript

Page 3: [82] Formatted **Ryan Powell** **11/02/2021 11:37:00**

Superscript

Page 3: [82] Formatted **Ryan Powell** **11/02/2021 11:37:00**

Superscript

Page 3: [82] Formatted **Ryan Powell** **11/02/2021 11:37:00**

Superscript

Page 3: [83] Formatted **Ryan Powell** **11/02/2021 11:37:00**

Superscript

Page 3: [83] Formatted **Ryan Powell** **11/02/2021 11:37:00**

Superscript

Page 3: [83] Formatted **Ryan Powell** **11/02/2021 11:37:00**

Superscript

Page 3: [83] Formatted **Ryan Powell** **11/02/2021 11:37:00**

Superscript

Page 3: [83] Formatted **Ryan Powell** **11/02/2021 11:37:00**

Superscript

Page 3: [83] Formatted **Ryan Powell** **11/02/2021 11:37:00**

Superscript

Page 3: [83] Formatted **Ryan Powell** **11/02/2021 11:37:00**
Superscript

Page 3: [83] Formatted **Ryan Powell** **11/02/2021 11:37:00**
Superscript

Page 3: [83] Formatted **Ryan Powell** **11/02/2021 11:37:00**
Superscript

Page 3: [84] Formatted **Ryan Powell** **11/02/2021 11:37:00**
Superscript

Page 3: [85] Formatted **Ryan Powell** **11/02/2021 11:20:00**
Left

Page 3: [86] Formatted **Ryan Powell** **11/02/2021 11:37:00**
Font: Palatino Linotype, Superscript

Page 3: [86] Formatted **Ryan Powell** **11/02/2021 11:37:00**
Font: Palatino Linotype, Superscript

Page 3: [86] Formatted **Ryan Powell** **11/02/2021 11:37:00**
Font: Palatino Linotype, Superscript

Page 3: [86] Formatted **Ryan Powell** **11/02/2021 11:37:00**
Font: Palatino Linotype, Superscript

Page 4: [87] Formatted **Ryan Powell** **04/02/2021 12:29:00**
Font: 9 pt

Page 4: [88] Formatted Table **Ryan Powell** **04/02/2021 12:36:00**
Formatted Table

Page 4: [89] Formatted **Ryan Powell** **04/02/2021 12:29:00**
Font: 9 pt

Page 4: [90] Formatted **Ryan Powell** **04/02/2021 12:29:00**
Font: 9 pt

Page 4: [91] Formatted **Ryan Powell** **04/02/2021 12:29:00**
Font: 9 pt

Page 4: [91] Formatted **Ryan Powell** **04/02/2021 12:29:00**
Font: 9 pt

Page 4: [92] Formatted **Ryan Powell** **04/02/2021 12:29:00**
Font: 9 pt

Page 4: [92] Formatted	Ryan Powell	04/02/2021 12:29:00

Font: 9 pt

Page 4: [93] Formatted	Ryan Powell	04/02/2021 12:29:00

Font: 9 pt

Page 4: [94] Formatted	Ryan Powell	04/02/2021 12:29:00

Font: 9 pt

Page 4: [95] Formatted	Ryan Powell	04/02/2021 12:29:00

Font: 9 pt

Page 4: [96] Formatted	Ryan Powell	04/02/2021 12:29:00

Font: 9 pt

Page 4: [97] Formatted	Ryan Powell	04/02/2021 12:29:00

Font: 9 pt

Page 4: [98] Formatted	Ryan Powell	04/02/2021 12:29:00

Font: 9 pt

Page 4: [99] Formatted	Ryan Powell	04/02/2021 12:29:00

Font: 9 pt

Page 4: [99] Formatted	Ryan Powell	04/02/2021 12:29:00

Font: 9 pt

Page 4: [100] Formatted	Ryan Powell	04/02/2021 12:29:00

Font: 9 pt

Page 4: [101] Formatted	Ryan Powell	04/02/2021 12:29:00

Font: 9 pt

Page 4: [102] Formatted	Ryan Powell	04/02/2021 12:29:00

Font: 9 pt

Page 4: [103] Formatted	Ryan Powell	04/02/2021 12:29:00

Font: 9 pt

Page 4: [104] Formatted	Ryan Powell	04/02/2021 12:29:00

Font: 9 pt

Page 4: [105] Formatted	Ryan Powell	04/02/2021 12:29:00

Font: 9 pt

Page 4: [106] Formatted	Ryan Powell	04/02/2021 12:29:00

Font: 9 pt

Page 4: [107] Formatted	Ryan Powell	04/02/2021 12:29:00

Font: 9 pt

Page 4: [107] Formatted	Ryan Powell	04/02/2021 12:29:00

Font: 9 pt

Page 4: [108] Formatted	Ryan Powell	04/02/2021 12:29:00

Font: 9 pt

Page 4: [108] Formatted	Ryan Powell	04/02/2021 12:29:00

Font: 9 pt

Page 4: [109] Formatted	Ryan Powell	04/02/2021 12:29:00

Font: 9 pt

Page 4: [109] Formatted	Ryan Powell	04/02/2021 12:29:00

Font: 9 pt

Page 4: [110] Formatted	Ryan Powell	04/02/2021 12:29:00

Font: 9 pt

Page 4: [111] Formatted	Ryan Powell	04/02/2021 12:29:00

Font: 9 pt

Page 4: [112] Formatted	Ryan Powell	04/02/2021 12:29:00

Font: 9 pt

Page 4: [113] Formatted	Ryan Powell	04/02/2021 12:30:00

Centred

Page 4: [114] Formatted	Ryan Powell	04/02/2021 12:29:00

Font: 9 pt

Page 4: [115] Formatted	Ryan Powell	04/02/2021 12:29:00

Font: 9 pt

Page 4: [116] Formatted	Ryan Powell	04/02/2021 12:29:00

Font: 9 pt

Page 4: [117] Formatted	Ryan Powell	04/02/2021 12:29:00

Font: 9 pt

Page 4: [118] Formatted	Ryan Powell	04/02/2021 12:29:00

Font: 9 pt

**Page 4: [119] Formatted**	**Ryan Powell**	**04/02/2021 12:29:00**

Font: 9 pt

**Page 4: [120] Formatted**	**Ryan Powell**	**04/02/2021 12:30:00**

Centred

**Page 4: [121] Formatted**	**Ryan Powell**	**04/02/2021 12:29:00**

Font: 9 pt

**Page 4: [122] Formatted**	**Ryan Powell**	**04/02/2021 12:29:00**

Font: 9 pt

**Page 4: [123] Formatted**	**Ryan Powell**	**04/02/2021 12:29:00**

Font: 9 pt

**Page 4: [124] Formatted**	**Ryan Powell**	**04/02/2021 12:30:00**

Centred

**Page 4: [125] Formatted**	**Ryan Powell**	**04/02/2021 12:30:00**

Centred

**Page 4: [126] Formatted**	**Ryan Powell**	**04/02/2021 12:30:00**

Centred

**Page 4: [127] Formatted**	**Ryan Powell**	**04/02/2021 12:30:00**

Centred

**Page 4: [128] Formatted**	**Ryan Powell**	**04/02/2021 12:29:00**

Font: 9 pt

**Page 4: [128] Formatted**	**Ryan Powell**	**04/02/2021 12:29:00**

Font: 9 pt

**Page 4: [129] Formatted**	**Ryan Powell**	**04/02/2021 12:30:00**

Centred

**Page 4: [130] Formatted**	**Ryan Powell**	**04/02/2021 12:29:00**

Font: 9 pt

**Page 4: [131] Formatted**	**Ryan Powell**	**04/02/2021 12:29:00**

Font: 9 pt

**Page 4: [132] Formatted**	**Ryan Powell**	**04/02/2021 12:29:00**

Font: 9 pt

**Page 4: [133] Formatted**	**Ryan Powell**	**04/02/2021 12:29:00**

Font: 9 pt

**Page 4: [134] Formatted**	**Ryan Powell**	**04/02/2021 12:29:00**

Font: 9 pt

**Page 4: [135] Formatted**	**Ryan Powell**	**04/02/2021 12:30:00**

Centred

**Page 4: [136] Formatted**	**Ryan Powell**	**04/02/2021 12:29:00**

Font: 9 pt

**Page 4: [137] Formatted**	**Ryan Powell**	**04/02/2021 12:29:00**

Font: 9 pt

**Page 4: [138] Formatted**	**Ryan Powell**	**04/02/2021 12:29:00**

Font: 9 pt

**Page 4: [139] Formatted**	**Ryan Powell**	**11/02/2021 13:12:00**

Left
