# Supplementary figures and images for: Differential Expression of BARD1 Isoforms in Melanoma
Source: Genes (Basel). 2021 Feb 23;12(2):320. doi: 10.3390/genes12020320 (PMC7927127; doi:10.3390/genes12020320)

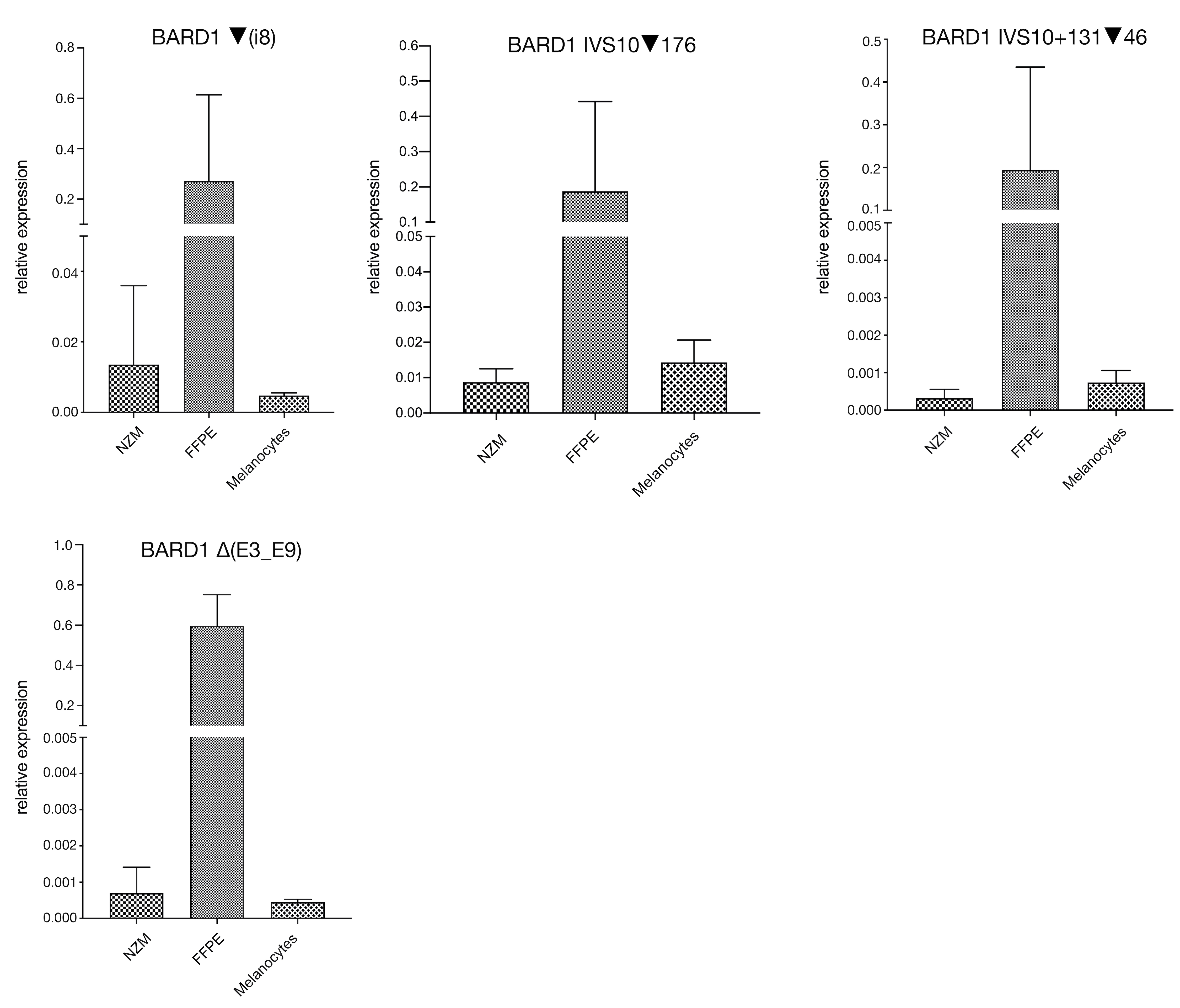

Supplement: Supplementary file 1 [file genes-12-00320-s001.zip › Suppl_Figure_S3.jpg]

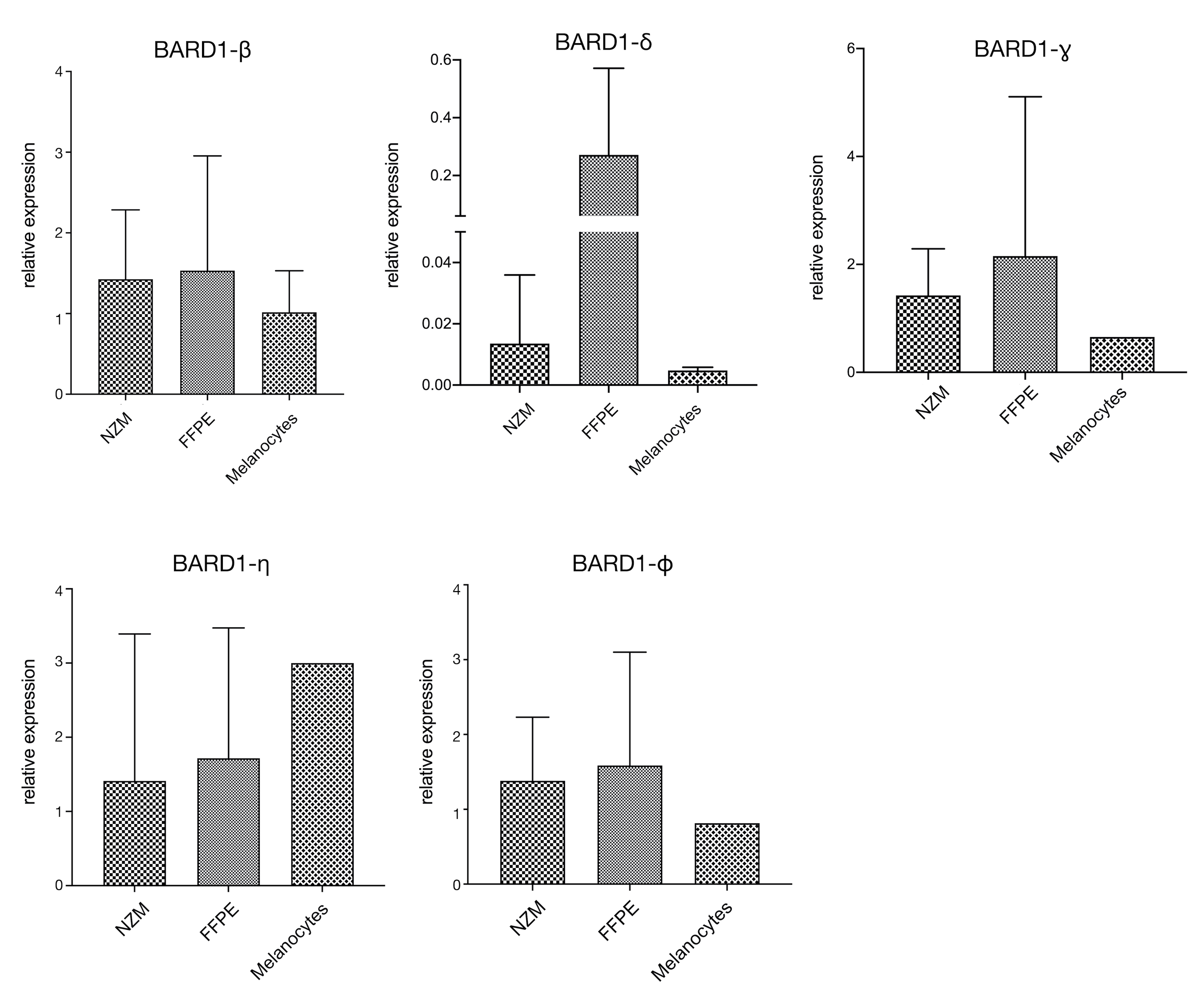

Supplement: Supplementary file 1 [file genes-12-00320-s001.zip › Suppl_Figure_S2.jpg]
